# Polyphyllin VII induces CTC anoikis to inhibit lung cancer metastasis through EGFR pathway regulation

**DOI:** 10.7150/ijbs.83682

**Published:** 2023-10-16

**Authors:** Zujun Que, Bin Luo, Pan Yu, Dan Qi, Wenji Shangguan, Panpan Wang, Jiajun Liu, Yan Li, He Li, Ronghu Ke, Erxi Wu, Jianhui Tian

**Affiliations:** 1 Institute of Oncology, Shanghai Municipal Hospital of Traditional Chinese Medicine, Shanghai University of Traditional Chinese Medicine (TCM), Shanghai, 200071, China.; 2 Institute of TCM Oncology, Longhua Hospital, Shanghai University of TCM, Shanghai, 200032, China.; 3 Clinical Oncology Center, Shanghai Municipal Hospital of TCM, Shanghai University of TCM, Shanghai, 200071, China.; 4 Department of Neurosurgery and Neuroscience Institute, Baylor Scott & White Health, Temple, TX 76508, USA.; 5 Department of Neurosurgery, Baylor College of Medicine, Temple, Texas 76508, USA.; 6 Department of TCM, Renji Hospital, School of Medicine, Shanghai Jiao Tong University, Shanghai, 200127, China.; 7 School of Medicine, Texas A&M University, College Station, TX 77843, USA.; 8 Irma Lerma Rangel School of Pharmacy, Texas A&M University, College Station, TX 77843, USA.; 9 LIVESTRONG Cancer Institutes and Department of Oncology, Dell Medical School, The University of Texas at Austin, Austin, TX 78712, USA.

**Keywords:** Lung cancer, Metastasis, Circulating tumor cells, Anoikis, Polyphyllin VII

## Abstract

Circulating tumor cells (CTCs) are cells that detach from the primary tumor and enter the bloodstream, playing a crucial role in the metastasis of lung cancer. Unfortunately, there is currently a lack of drugs specifically designed to target CTCs and prevent tumor metastasis. In this study, we present evidence that polyphyllin VII, a potent anticancer compound, effectively inhibits the metastasis of lung cancer by inducing a process called anoikis in CTCs. We observed that polyphyllin VII had significant cytotoxicity and inhibited colony formation, migration, and invasion in both our newly established cell line CTC-TJH-01 and a commercial lung cancer cell line H1975. Furthermore, we found that polyphyllin VII induced anoikis and downregulated the TrkB and EGFR-MEK/ERK signaling pathways. Moreover, activation of TrkB protein did not reverse the inhibitory effect of polyphyllin VII on CTCs, while upregulation of EGFR protein effectively reversed it. Furthermore, our immunodeficient mouse models recapitulated that polyphyllin VII inhibited lung metastasis, which was associated with downregulation of the EGFR protein, and reduced the number of CTCs disseminated into the lungs by inducing anoikis. Together, these results suggest that polyphyllin VII may be a promising compound for the treatment of lung cancer metastasis by targeting CTCs.

## Introduction

Lung cancer is the malignant tumor which poses a significant threat to human health and carries the highest morbidity and mortality among malignant tumors, resulting in substantial economic burdens 1, 2. It stands as the leading cause of cancer-related deaths worldwide for both men and women 3. In the United States, the overall 5-year survival rate for lung cancer patients is approximately 22.9%, plummeting to a mere 7% for patients with distant metastases 2. Metastasis is the primary cause of death for most lung cancer cases; however, the underlying mechanisms of tumor metastasis remain poorly understood, and approved drugs specifically targeting lung cancer metastasis are scarce 1. Thus, more knowledge of lung cancer metastasis and the discovery of new drugs that effectively target it are urgently needed.

Tumor cells that circulate in the bloodstream, known as circulating tumor cells (CTCs), act as a critical link between the primary tumor and metastasis. Extensive research has shown that CTCs can serve multiple purposes, including early diagnosis, assessment of metastasis risk, therapeutic monitoring, and personalized treatment for various types of tumors 4, 5. Moreover, in early-stage cancer patients, a higher number of CTCs is associated with poorer progression-free survival (PFS) and overall survival (OS) 6. The increase in CTC count during treatment can occur two years earlier than the detection of disease progression through imaging examinations 7, 8. Additionally, the frequency of genetic mutations in CTCs more closely resembles that of metastatic tumors rather than primary tumors 9. This evidence suggests that CTCs may act as initiators for tumor metastasis and could potentially be targeted for antimetastatic therapy 10. However, the development of drugs specifically targeting CTCs in the context of lung cancer remains limited.

*Paris polyphylla* Sm, a traditional Chinese medicine, is widely used in clinical practice due to its antitumor effects. It contains several active compounds, including polyphyllin I, II, III, V, VI, VII, and others 11. Among them, polyphyllin VII is the most effective compound. Previous studies have demonstrated that polyphyllin VII possesses broad-spectrum anticancer effects, including apoptosis induction and inhibition of metastasis in lung cancer, liver cancer, colorectal cancer, and other types of cancer cells 12-14. However, despite these notable findings, the specific involvement of polyphyllin VII in targeting CTCs for the suppression of tumor metastasis remains largely unknown. To address this knowledge gap, we conducted this study building upon our previous work, wherein we successfully established a lung cancer CTC line with metastatic capabilities, aptly named CTC-TJH-01 15, 16. The CTC-TJH-01 cells harbor a synonymous single nucleotide variant (SNV) (Q787Q) in exon 20 of the EGFR gene 15. By utilizing our established CTC line in conjunction with a widely employed lung cancer cell line, our investigation aimed to delve into the role of polyphyllin VII in CTC metastasis and unravel the potential molecular mechanisms underlying its actions.

## Materials and Methods

### Reagents and antibodies

Polyphyllin VII was procured from Beijing Solarbio Science & Technology Co., Ltd. (Beijing, China). The TrkB agonist 7,8-dihydroxyflavone (DHF), was obtained from Selleck (Shanghai, China). Recombinant human EGF protein was sourced from R&D Systems (Minneapolis, MN, USA). The anoikis detection kit was purchased from Biovision (Mountain View, CA, USA). Additionally, the Ras GTPase detection kit was purchased from Abcam (Cambridge, MA, UK). For antibody procurement, the following sources were utilized: EGFR, goat anti-mouse IgG-HRP, and donkey anti-rabbit IgG-HRP were obtained from Cell Signaling Technology (Danvers, MA, USA), while MMP-2, MMP-9, survivin, and GAPDH antibodies were procured from Proteintech (Wuhan, China). Furthermore, antibodies against MEK, p-MEK (Ser298), ERK1/2, p-ERK1/2 (Thr202/Thr185), BDNF, Ras, and Raf were sourced from Abcam (Cambridge, MA, UK).

### Cell culture

H1975 lung adenocarcinoma cells, 16HBE cells, and BEAS-2B normal lung epithelial cells were obtained from the Cell Bank of the Chinese Academy of Sciences (Shanghai, China). The human circulating lung tumor cell line CTC-TJH-01 was previously established and characterized by our laboratory 15, 16. Briefly, CTC-TJH-01 cells were cultured in F12K medium supplemented with 10% foetal bovine serum (Biological Industries, Israel) and penicillin-streptomycin (Gibco Life Technologies, Carlsbad, CA, USA). H1975, 16HBE and BEAS-2B cells were cultured in RPMI-1640 medium (Corning Cellgro, USA) containing 10% FBS and penicillin-streptomycin. All cells were maintained at 37°C in a humidified atmosphere with 5% CO_2_.

### Animals

Male NOD/SCID mice were procured from Shanghai Lingchang Biotechnology Co., Ltd. and maintained in the pathogen-free animal facility at Longhua Hospital, Shanghai University of Traditional Chinese Medicine. The mice were housed in accordance with the principles outlined in the Guide for the Care and Use of Laboratory Animals. All experimental procedures were approved by the Animal Research Committee of Longhua Hospital, Shanghai University of Traditional Chinese Medicine (Approval No: 2019-N027).

### Cell viability analysis

Cell viability was assessed using a cell counting kit-8 (CCK-8) assay (Dojindo, Shanghai, China) following previously described methods 17. In brief, cells were treated with various concentrations of polyphyllin VII for 24 h. Following a 3-h incubation with CCK-8 reagent, the absorbance values were measured at 450 nm, and survival curves were generated using GraphPad software.

### Cell clone formation assay

Clone formation assays were conducted following a previously established protocol 17. Briefly, 500 cells were seeded into individual wells of a 6-well plate and subsequently treated with varying concentrations of polyphyllin VII (0 μM, 0.5 μM, and 1 μM). The cells were cultured for a period of 12 days under suitable conditions. After the incubation period, colonies were stained with Giemsa and counted.

### Cell apoptosis analysis

CTC-TJH-01 and H1975 cells were treated with various concentrations of polyphyllin VII (0 μM, 0.5 μM, and 1 μM) and incubated for 24 h. Cell apoptosis was assessed using the Annexin V-FITC/PI apoptosis detection kit (BD Biosciences, CA, USA) and analyzed using a FACSVerse flow cytometer (BD Biosciences, CA, USA).

### Migration and invasion assays

Migration and invasion assays were performed as previously described 18. Briefly, 8×10^4^ cells suspended in 500 μl of serum-free medium were seeded into the upper chamber of a Transwell insert, while the lower chamber was filled with 750 μl of medium containing 20% FBS. Various concentrations of polyphyllin VII (0 μM, 0.5 μM, and 1 μM) were added to the cells. After a 16-h incubation period, non-migrated cells were gently removed using a cotton swab, and the migrated cells in the Transwell chamber were fixed and stained with Giemsa solution. Subsequently, under an inverted microscope, five random fields were photographed and counted at a magnification of 100×. For the cell invasion assay, Transwell inserts coated with Matrigel were used.

### Western blot analysis

Western blotting was performed following a previously described method 17. Briefly, 3×10^5^ cells were seeded into individual wells of a 6-well plate and treated with different concentrations of polyphyllin VII (0 μM, 0.5 μM, and 1 μM) for 24 h. The cells were then lysed and centrifuged at 12,000 rpm for 30 minutes at 4°C. The protein content in the lysates was extracted and quantified using the BCA assay. Subsequently, 40 μg of the proteins were loaded onto 5% to 12.5% SDS polyacrylamide gels and subjected to electrophoresis. The separated proteins were then transferred to a polyvinylidene difluoride membrane. The membranes were blocked using skim milk and subsequently incubated with specific primary antibodies, followed by appropriate secondary antibodies. Immunoreactive bands were visualized using an ECL kit (Bio-Rad, CA, USA). The density of the bands was analyzed using ImageJ software (Bio-Rad, CA, USA).

### Anoikis assays

Anoikis assays were conducted following a previously established protocol 18. In brief, 1×10^5^ cells were seeded into 24-well plates that were either coated with poly-HEMA (to promote cell detachment) or left uncoated. The cells were then treated with different concentrations of polyphyllin VII (0 μM, 0.5 μM, and 1 μM) for 24 hours. Subsequently, each well was supplemented with a solution containing Calcein AM and ethidium homodimer-1 (EthD-1) 19, and the plates were incubated for 45 minutes at 37°C. An inverted fluorescence microscope was used to observe and capture images of cell survival or anoikis. Live cells were visualized using green fluorescence from Calcein AM, while cells undergoing anoikis were stained with red fluorescence from EthD-1.

### Ras GTPase assays

Ras GTPase assays were conducted following the experimental manual's instructions. In summary, 3×10^5^ cells were seeded into 6-well plates and treated with varying concentrations of polyphyllin VII (0 μM, 0.5 μM, and 1 μM) for 24 h. After washing the cells with ice-cold PBS, complete lysis/binding buffer was added to extract the proteins, and their protein content was measured using a Bradford-based assay. Subsequently, the activation of Ras was quantified using ELISA, adhering to the guidelines provided in the experimental manual.

### Lung metastasis assays

CTC-TJH-01 cells (1×10^6^ cells/100 μl) were injected into the tail veins of 6-week-old male NOD-SCID mice, following a previously established protocol 15, 16. Subsequently, 10 mice were randomly assigned to each experimental group. The next day, the mice were administered either polyphyllin VII (10 mg/kg/d) or an equivalent dose of normal saline via intraperitoneal injection. This administration was repeated three times a week for a total duration of four weeks. At the end of 10 weeks, the mice were sacrificed, and their lungs were removed. Pulmonary metastatic nodules were visually examined, counted, and photographed under a dissecting microscope. Hematoxylin and eosin (H&E) staining as well as immunohistochemistry were performed on the lung samples.

### Quantification of CTCs in NOD-SCID mice

NOD-SCID mice were initially administered either polyphyllin VII (10 mg/kg/day) or an equivalent dose of normal saline via intraperitoneal injection. Subsequently, the mice were injected with CTC-TJH-01-GFP cells (1×10^6^ cells/100 μl) through the tail vein. At 24 h post-injection, the mice were euthanized, and the blood and lung samples were collected. CTCs displaying green fluorescence were quantified using a fluorescence microscope. The lungs were fixed in 4% paraformaldehyde and embedded in paraffin blocks. Lung sections were then examined under a fluorescence microscope for further analysis.

### Immunohistochemistry assays

The immunohistochemistry assays were performed as previously described 20. The lungs were fixed in 4% paraformaldehyde and subsequently embedded in paraffin blocks. Thin sections of the lung tissue were prepared and stained with hematoxylin and eosin (H&E) for general histological examination. Additionally, anti-human antibodies targeting EGFR were used for immunohistochemical staining. The stained slides were scanned at a magnification of 100× using a KF-PRO-020 digital slide scanner (Ningbo Jiangfeng Biological Information Technology Co., Ltd.).

### Statistical analysis

All experiments were performed at least in triplicate (n = 3). Data were presented by the mean ± standard deviation (SD) or the standard error of the mean (SEM). Statistical analyses were conducted using GraphPad Prism 9.0 software (GraphPad, San Diego, CA, USA) or R package rstatix (CRAN R 4.3.1, RStudio 2023.06.0+421). The differences between two groups were assessed using parametric Student's t-test or nonparametric Mann-Whitney-Wilcoxon test. For analyses involving multiple groups, one-way analysis of variance (ANOVA) was applied. The levels of statistical significance were set at *P<0.05, **P<0.01, and ***P<0.001.

## Results

**Polyphyllin VII inhibits the proliferation of lung cancer CTCs**

**Figure 1:**
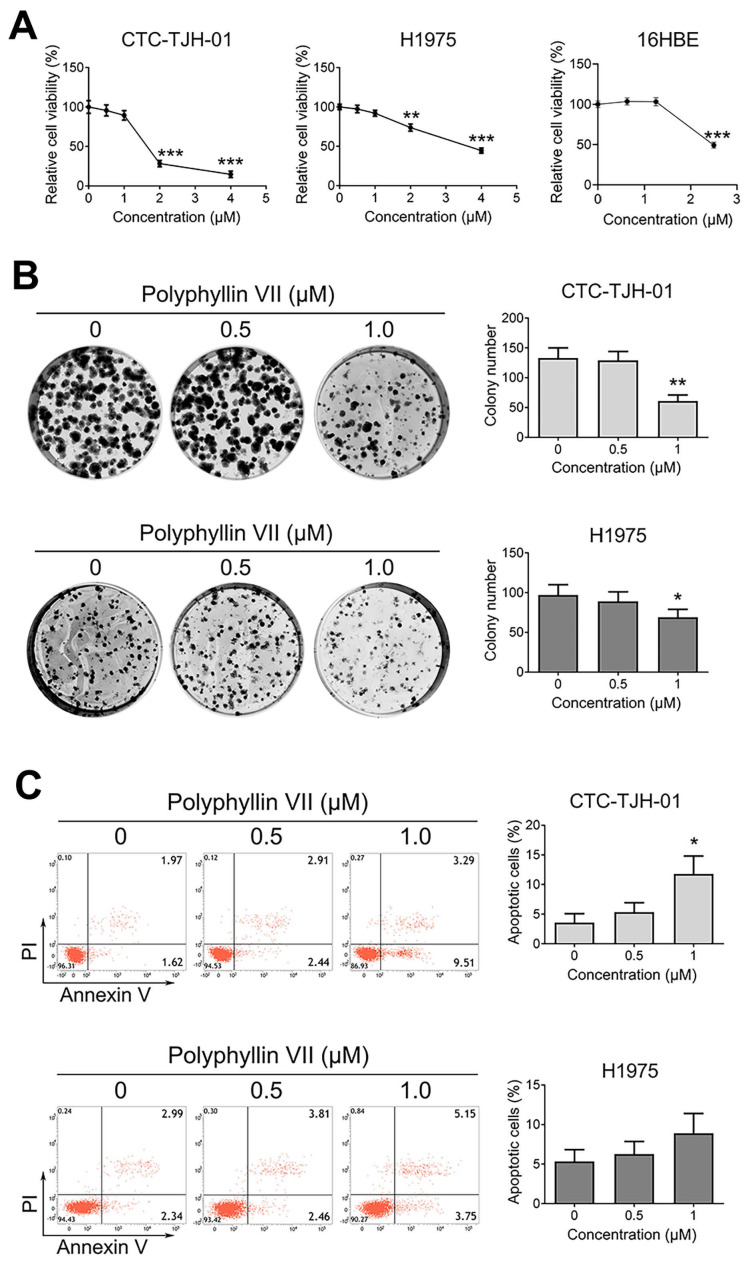
** Polyphyllin VII exhibits anti-proliferative effects on CTCs in lung cancer**.** (A)** CTC-TJH-01, H1975, and 16HBE cells were treated with varying concentrations of polyphyllin VII for 24 h, after which the cell viability was measured using a CCK-8 assay. **(B)** The colony-forming ability of CTC-TJH-01 and H1975 cells was assessed by a colony forming assay. **(C)** Annexin V-FITC/PI staining was performed to detect apoptotic cells. The data presented represent the mean ± SD of three independent experiments, and each bar represents the respective results. Statistical significance is denoted as *P < 0.05; **P < 0.01; and ***P < 0.001.

To assess the antiproliferative effects of polyphyllin VII, we conducted experiments using human circulating lung tumor CTC-TJH-01 cells, human lung cancer H1975 cells, and normal lung epithelial 16HBE cells. A CCK-8 assay was employed to evaluate cell proliferation. As depicted in Figure 1A, polyphyllin VII significantly inhibited CTC-TJH-01, H1975, and 16HBE cell proliferation. Subsequently, we selected concentrations (0.5 μM and 1 μM) of polyphyllin VII that exhibited no evident cytotoxic effects on 16HBE normal lung epithelial cells for further investigations. Furthermore, treatment with 1 μM polyphyllin VII notably suppressed the size and number of CTC-TJH-01 and H1975 cells, as illustrated in Figure 1B. Additionally, the apoptosis assay revealed that polyphyllin VII induced apoptosis in CTC-TJH-01 cells at a concentration of 1 μM, while no significant apoptosis was observed in H1975 cells (Figure 1C). These findings collectively indicate that polyphyllin VII effectively inhibits the proliferation of CTC-TJH-01 and H1975 cells while displaying no detrimental effects on normal 16HBE cells.

### Polyphyllin VII impedes the migration and invasion of lung cancer CTCs

**Figure 2:**
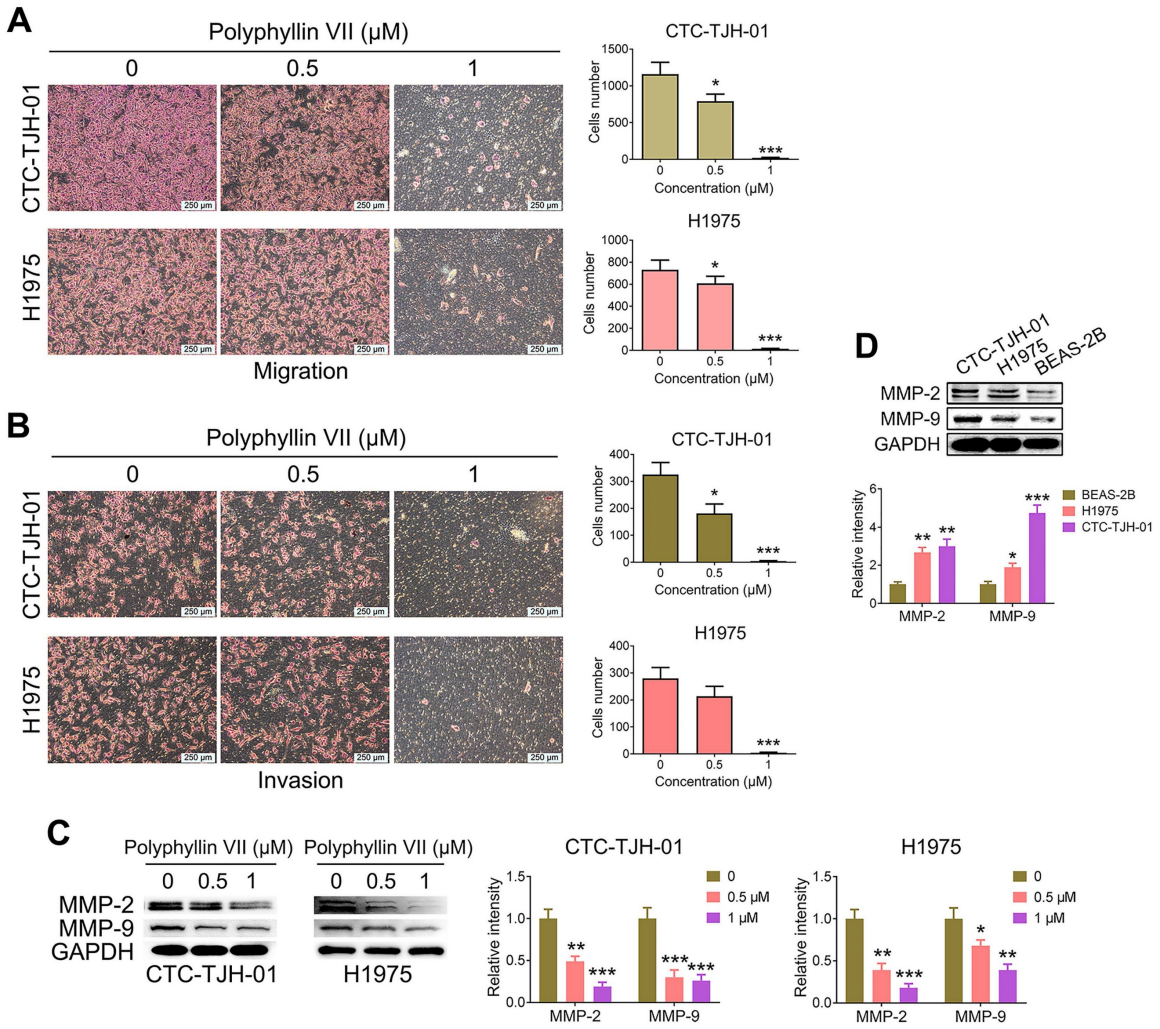
** Polyphyllin VII inhibits the migration and invasion of lung cancer CTCs. (A)** The migration capacity of CTC-TJH-01 and H1975 cells was measured using Transwell assays after treatment with polyphyllin VII (0 μM, 0.5 μM, and 1 μM) for 16 h. The scale bar represents 250 μm. **(B)** The invasion capacity of CTC-TJH-01 and H1975 cells was measured using Transwell assays with Matrigel invasion chambers after polyphyllin VII (0 μM, 0.5 μM, and 1 μM) treatment for 16 h. Scale bar: 250 μm. **(C)** CTC-TJH-01 and H1975 cells were incubated with polyphyllin VII (0 μM, 0.5 μM, and 1 μM) for 24 h. A western blot assay was used to measure the protein expression levels of MMP-2 and MMP-9 in CTC-TJH-01 and H1975 cells. GAPDH was used as an internal control. **(D)** A western blot assay was used to measure the protein expression levels of MMP-2 and MMP-9 in CTC-TJH-01, H1975 and BEAS-2B cells. GAPDH was used as an internal control. Each bar represents the mean ± SD of three separate experiments. Statistical significance is indicated as *P < 0.05; **P < 0.01; ***P < 0.001.

To investigate the impact of polyphyllin VII on lung cancer CTC migration and invasion, we conducted Transwell assays. As depicted in Figure 2A, polyphyllin VII exerted a significant inhibitory effect on the migration of CTC-TJH-01 and H1975 cells. Similarly, the invasion assay demonstrated a substantial suppression of invasive cells upon treatment with polyphyllin VII, as illustrated in Figure 2B. Furthermore, we assessed the expression of MMP-2 and MMP-9 proteins, which play crucial roles in cell migration and invasion. Figure 2C reveals that polyphyllin VII treatment led to a remarkable downregulation of MMP-2 and MMP-9 protein levels in both CTC-TJH-01 and H1975 cells. Importantly, we observed that CTC-TJH-01 cells exhibited higher expression of MMP-2 and MMP-9 proteins than H1975 cells (Figure 2D). Collectively, these findings demonstrate that polyphyllin VII can inhibit the metastasis of lung cancer CTCs *in vitro*.

### Polyphyllin VII induces anoikis of lung cancer CTCs

**Figure 3:**
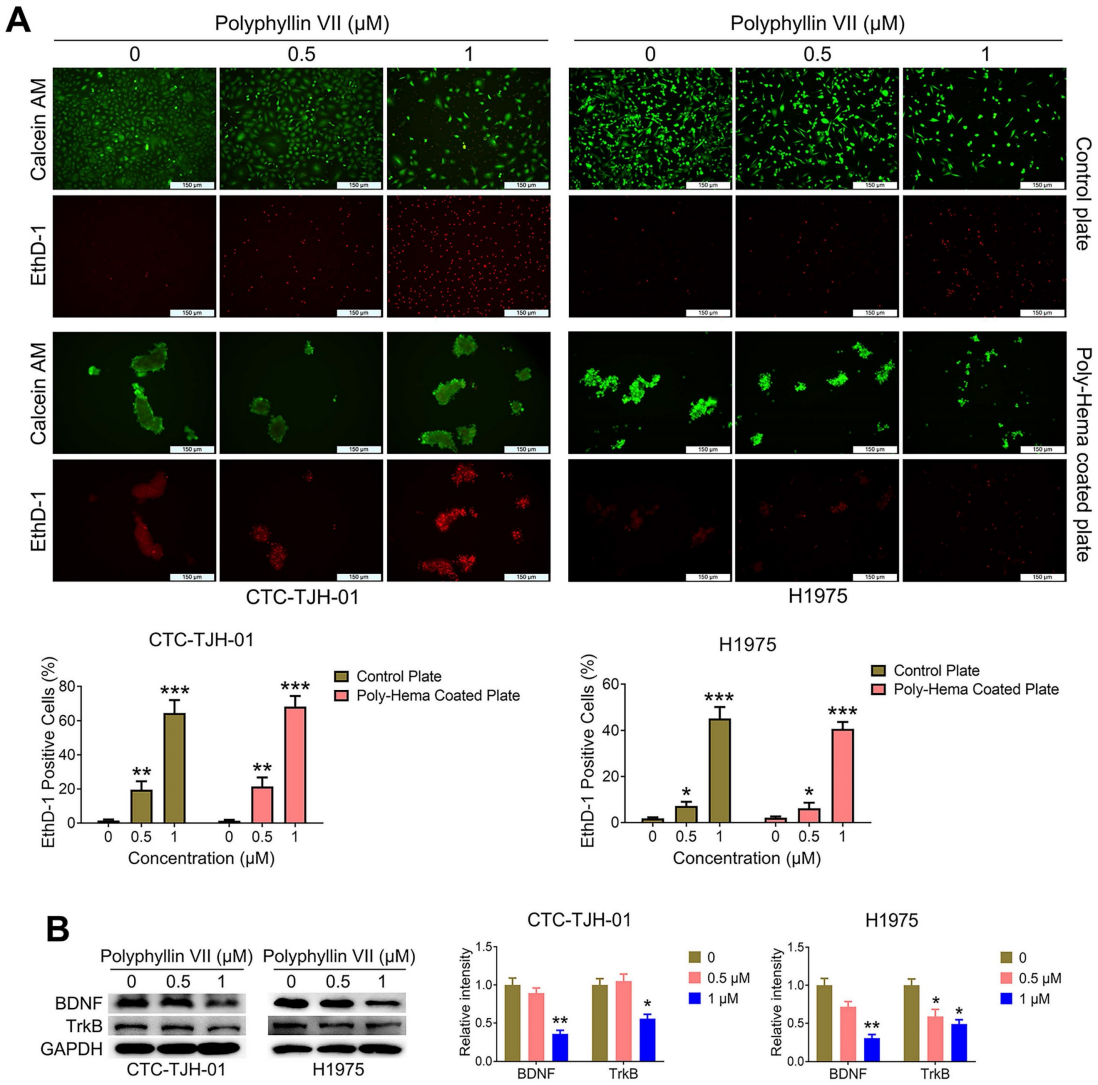
** Polyphyllin VII induces anoikis of lung cancer CTCs. (A)** An anoikis assay was used to measure the anoikis levels in CTC-TJH-01 and H1975 cells after incubation with polyphyllin VII (0 μM, 0.5 μM, and 1 μM) for 24 h. Scale bar: 150 μm. **(B)** Western blot assay was employed to assess the protein expression levels of BDNF and TrkB in CTC-TJH-01 and H1975 cells. GAPDH served as an internal control. Each bar represents the mean ± SD of three independent experiments. Statistical significance is indicated as *P < 0.05; **P < 0.01; ***P < 0.001.

To unravel the underlying mechanisms of polyphyllin VII's anti-metastatic effects on lung cancer CTCs, we employed an anoikis detection kit to assess its impact on CTC anoikis. As depicted in Figure 3A, polyphyllin VII significantly induced anoikis in CTC-TJH-01 and H1975 cells. Furthermore, we examined the expression of TrkB proteins, a known marker for anoikis, and found that polyphyllin VII led to a significant downregulation of TrkB protein levels in both CTC-TJH-01 and H1975 cells (Figure 3B). Additionally, we investigated the expression of brain-derived neurotrophic factor (BDNF), which is capable of activating TrkB. Encouragingly, polyphyllin VII treatment resulted in a significant downregulation of BDNF expression, further supporting the involvement of the BDNF/TrkB pathway in polyphyllin VII-induced anoikis. These findings collectively suggest that polyphyllin VII may induce anoikis in lung cancer CTCs by modulating the BDNF/TrkB pathway, subsequently impeding their metastatic potential.

### Polyphyllin VII induces anoikis and inhibits the migration of lung cancer CTCs via a mechanism other than the BDNF/TrkB axis

**Figure 4:**
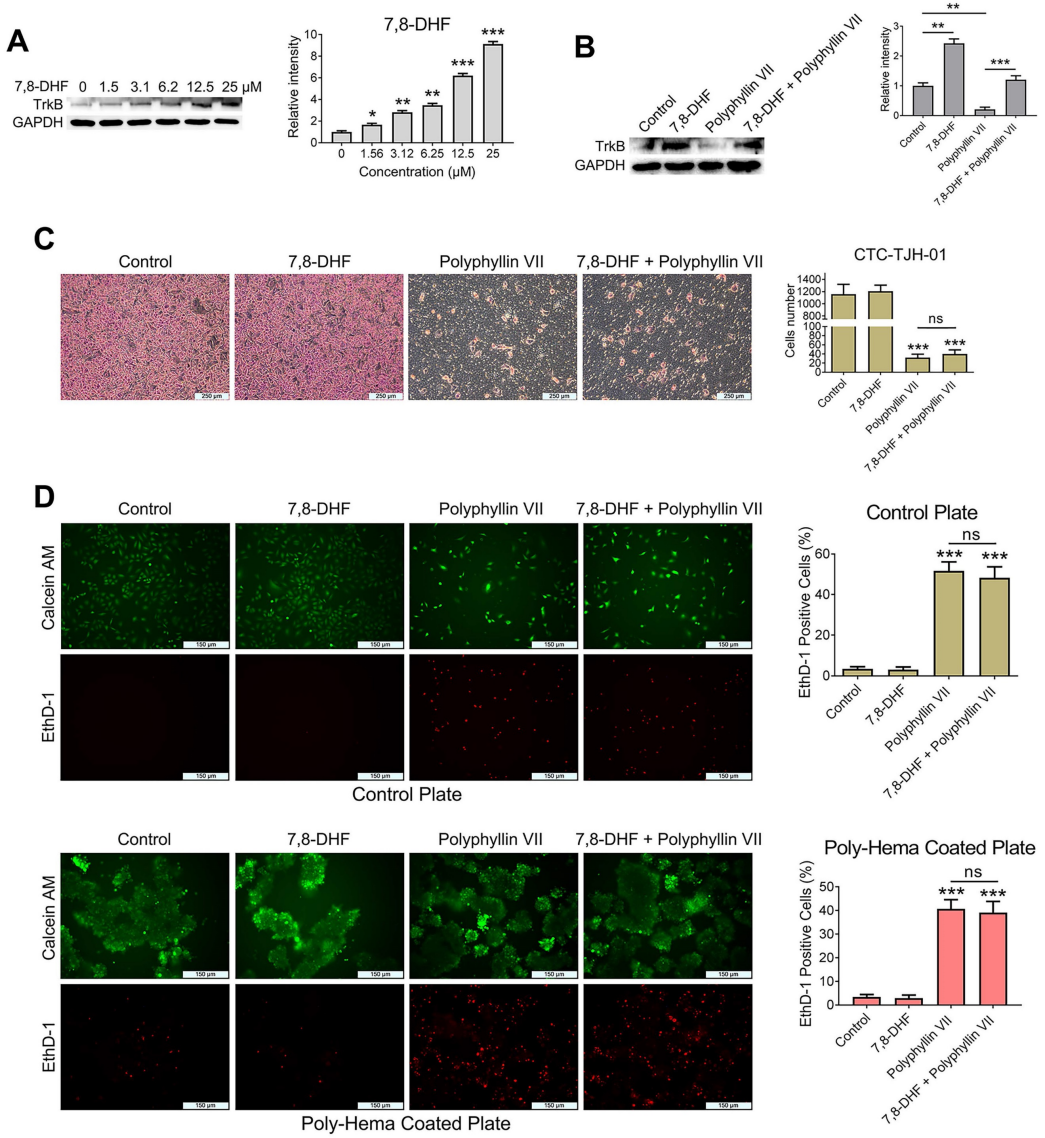
** Polyphyllin VII induces anoikis and inhibits the migration of lung cancer CTCs via a mechanism other than the BDNF/TrkB axis. (A)** CTC-TJH-01 cells were treated with varying concentrations of 7,8-DHF for 24 h, and the expression of TrkB protein was detected by western blot. GAPDH was used as an internal control. **(B)** CTC-TJH-01 cells were incubated with polyphyllin VII (1 μM), 7,8-DHF (10 μM) or a combination of both for 24 h. The protein expression levels of TrkB in CTC-TJH-01 cells were measured using western blot analysis. GAPDH was used as an internal control. **(C)** The migration ability of CTC-TJH-01 cells was measured using transwell assays after polyphyllin VII (1 μM), 7,8-DHF (10 μM) or their combination treatment for 16 h. Scale bar: 250 μm. **(D)** An anoikis assay was used to measure the anoikis levels in CTC-TJH-01 cells after incubation with polyphyllin VII (1 μM), 7,8-DHF (10 μM) or their combination for 24 h. Scale bar: 150 μm. Each bar represents the mean ± SD of three separate experiments. Statistical significance is indicated as *P < 0.05; **P < 0.01; ***P < 0.001.

For CTCs to successfully establish themselves in other “hot” niches, they should overcome a process known as anoikis, a type of apoptosis triggered when cells lose their adhesion to the extracellular matrix (ECM). Resistance to anoikis plays a crucial role in facilitating CTC metastasis. 21, 22. To gain further insights into the molecular mechanism underlying polyphyllin VII-induced CTC anoikis and its inhibitory effects on CTC metastasis, we conducted experiments combining the TrkB agonist 7,8-DHF with polyphyllin VII in CTC-TJH-01 cells. We observed that 7,8-DHF dose-dependently upregulated the expression of TrkB protein in CTC-TJH-01 cells (Figure 4A). Remarkably, when 7,8-DHF was co-administered with polyphyllin VII, it reversed the inhibitory effect of polyphyllin VII on TrkB protein in CTC-TJH-01 cells (Figure 4B). However, the results obtained from cell migration and anoikis assays revealed that 7,8-DHF failed to reverse the inhibitory effects of polyphyllin VII on CTC-TJH-01 cell migration and anoikis (Figure 4C&D). These findings strongly indicate that TrkB is not a regulatory protein mediating the induction of anoikis and the inhibition of CTC migration by polyphyllin VII. Taken together, these results suggest that polyphyllin VII exerts its effects on inducing anoikis and inhibiting CTC migration through a mechanism independent of the TrkB pathway. Alternative pathways or factors are likely involved in mediating the observed effects of polyphyllin VII on CTCs.

### Polyphyllin VII inhibits the EGFR-MEK/ERK signaling cascade in lung cancer CTCs

**Figure 5:**
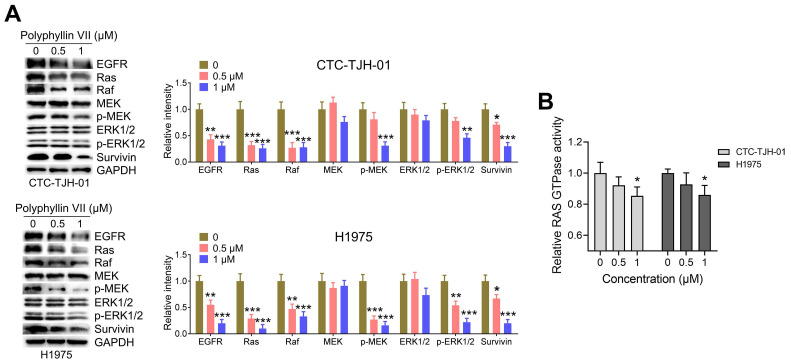
** Polyphyllin VII blocks the EGFR-MEK/ERK pathway in lung cancer CTCs.** CTC-TJH-01 and H1975 cells were incubated with polyphyllin VII (0 μM, 0.5 μM, and 1 μM) for 24 h. **(**A**)** Protein expression levels of EGFR, Ras, Raf, MEK, p-MEK, ERK1/2, p-ERK1/2, and survivin in CTC-TJH-01 and H1975 cells were determined using a western blot assay. GAPDH served as an internal control. **(B)** Ras activation levels in CTC-TJH-01 and H1975 cells were measured using a Ras GTPase assay. Each bar represents the mean ± SD of three independent experiments. Statistical significance is indicated as *P < 0.05; **P < 0.01; ***P < 0.001.

EGFR signaling is known to play a significant role in anoikis 21, 23, 24, making it an important pathway to explore. In order to gain insights into the potential molecular mechanism, we investigated the effects of polyphyllin VII on the EGFR pathways in CTC-TJH-01 and H1975 cells. As shown in Figure 5A, polyphyllin VII exhibited a significant downregulation of EGFR expression in both CTC-TJH-01 and H1975 cells. Furthermore, downstream proteins including Ras, Raf, p-MEK, p-ERK1/2, and survivin were also significantly downregulated upon polyphyllin VII treatment (Figure 5A). Additionally, Ras GTPase, a key component of the EGFR signaling pathway, was significantly downregulated by polyphyllin VII in both CTC-TJH-01 and H1975 cells (Figure 5B). These data strongly suggest that polyphyllin VII has the potential to induce anoikis and inhibit CTC migration by regulating the EGFR-MEK/ERK signaling pathway. The downregulation of EGFR and its downstream effectors, as well as the modulation of Ras GTPase, highlight the involvement of this pathway in mediating the observed effects of polyphyllin VII on CTCs.

### Polyphyllin VII induces anoikis and inhibits the migration of CTCs by downregulating the EGFR pathway

**Figure 6:**
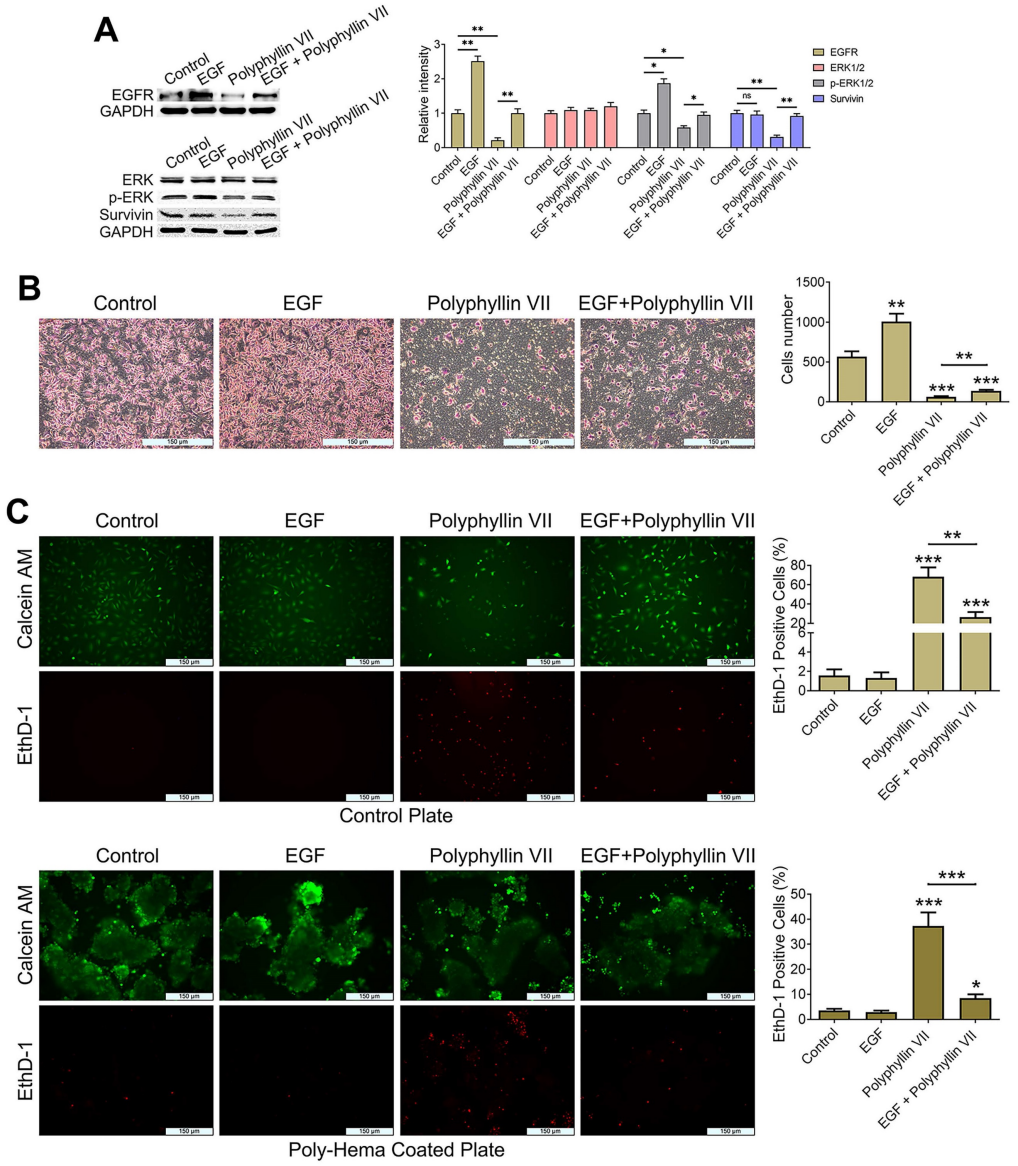
** Polyphyllin VII inhibits CTC migration and induces anoikis via the EGFR pathway. (A)** CTC-TJH-01 cells were incubated with polyphyllin VII (1 μM), EGF (1 ng/mL) or a combination of both for 24 h. Protein expression levels of EGFR, ERK1/2, p-ERK1/2, and survivin in CTC-TJH-01 cells were measured using a western blot assay. GAPDH served as an internal control. **(B)** Transwell assay was performed to assess CTC-TJH-01 cell migration after treatment with polyphyllin VII (1 μM), EGF (1 ng/mL), or a combination of both. Representative images show migrated cells, and the scale bar represents 150 μm. **(C)** Anoikis assays were conducted to measure the levels of anoikis in CTC-TJH-01 cells following incubation with polyphyllin VII (1 μM), EGF (1 ng/mL), or a combination of both for 24 hours. Representative images demonstrate the extent of anoikis, and the scale bar represents 150 μm. Each bar represents the mean ± SD of three independent experiments. Statistical significance is indicated as *P < 0.05; **P < 0.01; ***P < 0.001.

**Figure 7:**
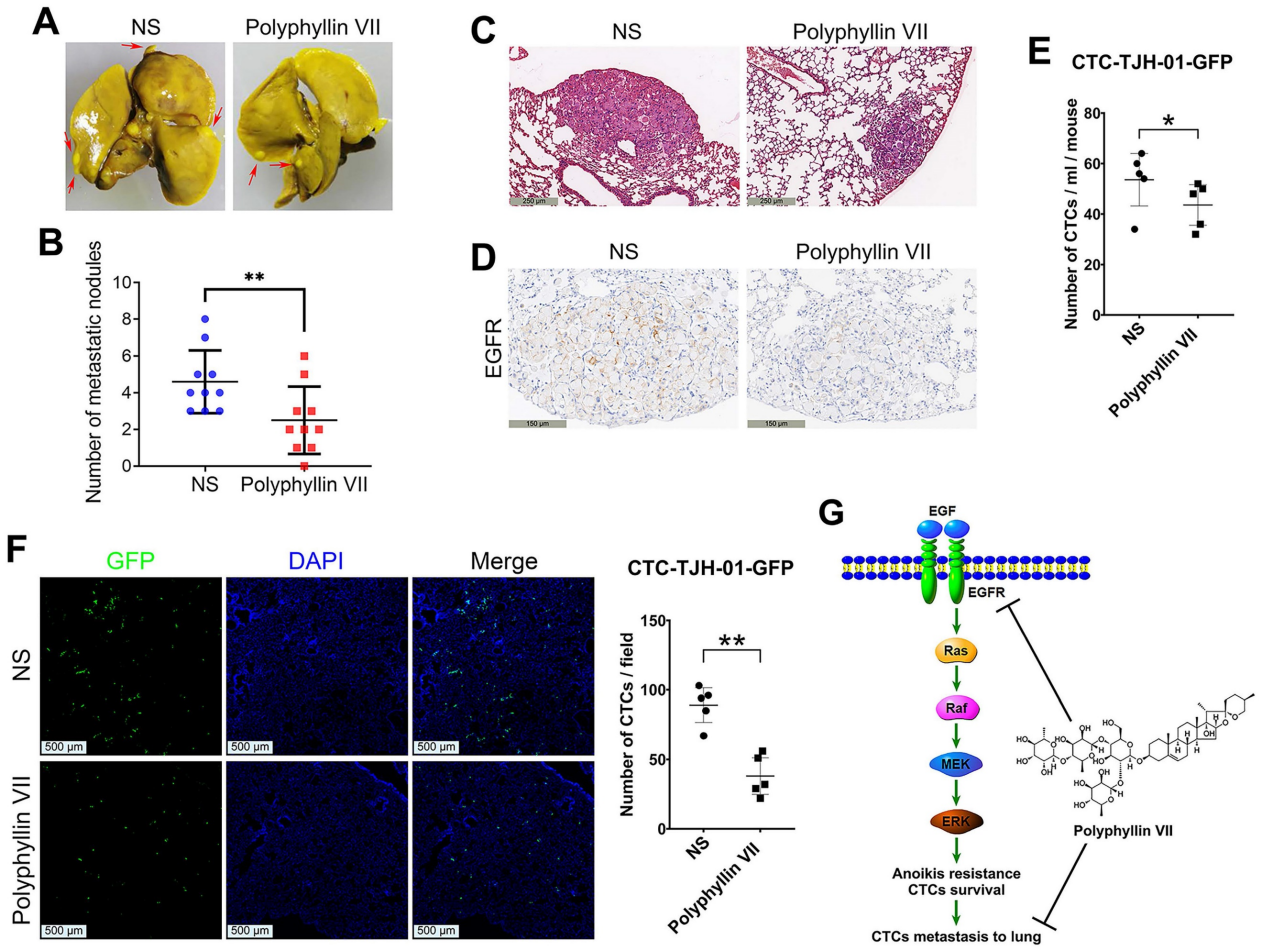
** Polyphyllin VII inhibits lung metastasis of CTCs *in vivo*. (A)** Representative images of lungs from NOD-SCID mice 10 weeks after CTC-TJH-01 cell injection. **(B)** Quantification of the number of pulmonary metastatic nodules in mice treated with polyphyllin VII (n=10). **(C)** H&E staining was performed to observe the morphology of pulmonary metastatic nodules. The scale bar represents 250 μm. **(D)** Immunohistochemistry was used to detect the expression of EGFR protein in pulmonary metastatic nodules. The scale bar represents 150 μm. **(E)** Quantification of CTCs in mouse peripheral blood at 24 h after CTC-TJH-01 cells injection in NOD/SCID mice treated with polyphyllin VII (10 mg/kg/d) (n=5/group).** (F)** Representative images and quantification of CTCs in mouse lungs 24 hours after CTC-TJH-01 cell injection in NOD/SCID mice treated with polyphyllin VII (10 mg/kg/d) (n=5/group). The scale bar represents 500 μm. **(G)** Diagram illustrating the inhibitory mechanism of polyphyllin VII on lung cancer metastasis by blocking the EGFR-MEK/ERK pathway and inducing CTC anoikis. Statistical significance is indicated as *P < 0.05; **P < 0.01; ***P < 0.001.

To further investigate the involvement of the EGFR pathway in polyphyllin VII-induced CTC anoikis, we employed EGF protein to upregulate the expression of EGFR protein in CTC-TJH-01 cells. Subsequently, we treated these cells with a combination of EGF and polyphyllin VII. As depicted in Figure 6A, EGF protein significantly increased the expression of EGFR protein in CTC-TJH-01 cells, effectively reversing the inhibitory effect of polyphyllin VII on EGFR protein levels. Moreover, EGF protein also reversed the inhibitory effect of polyphyllin VII on p-ERK and survivin proteins (Figure 6A), further suggesting the involvement of the EGFR pathway in polyphyllin VII-induced effects. Furthermore, we observed that EGF protein promoted the migration of CTC-TJH-01 cells and partially reversed the inhibitory effect of polyphyllin VII on cell migration in the combined treatment group (Figure 6B). A similar pattern was also observed in the anoikis assay, where the combination of EGF with polyphyllin VII significantly reversed the anoikis induction by polyphyllin VII in CTC-TJH-01 cells (Figure 6C). Additionally, immunohistochemical analysis revealed a significant inhibition of EGFR protein expression in tumor tissues following polyphyllin VII treatment (Figure 7D). These findings collectively indicate that polyphyllin VII exerts its inhibitory effect on CTC metastasis by downregulating the expression of EGFR protein (Figure 7G). Therefore, polyphyllin VII may suppress CTC metastasis by targeting the EGFR signaling pathway and inducing CTC anoikis.

### Polyphyllin VII suppresses lung metastasis of CTCs

To further validate our *in vitro* findings demonstrating the antimetastatic effects of polyphyllin VII, we conducted* in vivo* experiments using male NOD-SCID mice. CTC-TJH-01 cells were injected into the mice via the tail vein, and polyphyllin VII was administered as a treatment. Remarkably, the polyphyllin VII treatment group exhibited a significantly lower number of pulmonary metastatic nodules compared to the control group treated with normal saline (Figure 7A&B). Histological analysis through H&E staining further revealed that polyphyllin VII treatment reduced the density of metastatic nodules (Figure 7C&D). Additionally, an examination of CTCs in both peripheral blood and lung tissues of the mice revealed a significant reduction after administration of polyphyllin VII (Figure 7E&F). These findings suggest that polyphyllin VII specifically targets CTCs to exerting an inhibitory effect on lung metastasis. Taken together, these results provide compelling evidence that polyphyllin VII can effectively suppress the lung metastasis of CTCs. This effect is likely mediated through the inhibition of the EGFR signaling pathway, leading to the induction of CTC anoikis (Figure 7G).

## Discussion

In this study, we investigated the effects of polyphyllin VII on lung cancer metastasis using CTCs and lung adenocarcinoma cells. Our findings demonstrate that polyphyllin VII effectively reduces metastasis by inducing anoikis through the downregulation of the EGFR pathway. These results provide novel insights into the antimetastatic properties of polyphyllin VII.

Polyphyllin VII, a steroidal saponin derived from Paris polyphylla Sm., possesses potent anticancer activity against various human cancer cell lines 13, 14. Previous studies have reported the inhibitory effects of polyphyllin VII on the proliferation and apoptosis induction of different types of cancer cells, including human lung cancer cells (A549 and NCI-H1299 cells), human oral cancer cells (SAS and OECM-1 cells), human liver cancer cells (HepG2 cells), human nasopharyngeal cancer cells (HONE-1 and NPC-039 cells) and human colorectal cancer cells (HCT-116 and HT-29 cells) 12-14, 25, 26. In our study, we utilized our own established human lung cancer cell line CTC-TJH-01, which possesses metastatic capabilities, as well as the commercial lung cancer cell line H1975, to investigate the anticancer efficacy of polyphyllin VII. Our results demonstrate significant cytotoxic effects of polyphyllin VII on the proliferation of these cell lines. Notably, CTC-TJH-01 cells, derived from peripheral blood, have the advantage of acquiring anoikis resistance, which promotes CTC metastasis. Additionally, CTC-TJH-01 cells exhibit drug resistance, stem cell-like properties, immune evasion, tumorigenicity, and the ability to induce lung metastasis in immunodeficient mice, as previously reported 16. Moreover, CTC-TJH-01 cells harbor a synonymous SNV (Q787Q) in exon 20 of EGFR, while H1975 cells possess missense mutations at codon 858 (L858R) in exon 21 and at position 790 in exon 20 (T790M). We noticed that polyphyllin VII showed a cytotoxic effect at a concentration of 2.5 μM on normal lung epithelial 16HBE cells. Therefore, we carefully selected drug concentrations that exhibited no obvious cytotoxic effect on 16HBE cells for antimetastatic studies, namely concentrations presenting the best therapeutic index. We found that polyphyllin VII at a concentration of 1 μM could still significantly inhibit the clonal formation of CTC-TJH-01 and H1975 cells and caused approximately 10% of the cells to undergo apoptosis. In addition, polyphyllin VII significantly inhibited the migration and invasion of CTC-TJH-01 and H1975 cells *in vitro* and significantly downregulated the expression of MMP-2 and MMP-9 proteins in CTC-TJH-01 and H1975 cells. These observations are consistent with previous studies demonstrating the inhibitory effect of polyphyllin VII on the migration of HCT116 and HepG2 cells *in vitro* 12, 13.

Anoikis is a form of programmed cell triggered by the loss of cell adhesion to the ECM and plays a crucial role in tumor metastasis 27. In primary tumors, MMPs play a crucial role in promoting anoikis by cleaving basement membrane survival signals, such as cadherins and integrins. By disrupting these signals, MMPs contribute to the detachment and subsequent cell death of tumor cells when they lose contact with the extracellular matrix 28, 29. Additionally, MMPs can cleave BDNF, which induces CTCs to acquire resistance to anoikis through its receptor TrkB. The cleavage of BDNF by MMPs can disrupt this signaling pathway and further enhance the sensitivity of CTCs to undergo anoikis. Furthermore, MMPs facilitate the metastasis process by promoting the degradation of extracellular matrix components, allowing CTCs to extravasate from blood vessels into target organs 30. In line with the literature, our data revealed that polyphyllin treatment led to a decrease in the protein levels of MMP-2 and MMP-9 (Figure 2C), indicating a polyphyllin VII-induced downregulation of MMP-2/9 in lung CTCs. Due to the influence of circulating shear force, most CTCs in peripheral blood are in an anoikis state, while a small number of CTCs with anoikis resistance will spread to distant target organs and eventually form metastases 31. Tumor cells acquire anoikis resistance by activating survival signaling pathways and inhibiting apoptosis signaling 32. The BDNF/TrkB signaling pathway is involved in the regulation of anoikis 33, 34. Recently, Li Tao et al. found that activation of the BDNF/TrkB pathway could promote the migration and invasion of prostate cancer cells via induction of anoikis resistance 35. In our study, we demonstrate that polyphyllin VII induces significant anoikis in CTC-TJH-01 and H1975 cells, concomitant with a significant downregulation of the BDNF/TrkB axis. However, the activation of TrkB by the TrkB agonist, 7,8-DHF, did not reverse the polyphyllin VII-induced anoikis or inhibit the migration of lung cancer cells. These findings suggest that the induction of anoikis by polyphyllin VII in CTC-TJH-01 cells is independent of the BDNF/TrkB pathway.

In addition to the BDNF/TrkB axis, the EGFR signaling pathway is associated with tumor cell proliferation, angiogenesis, tumor invasion and metastasis, and apoptosis inhibition 36. Also, the initiation and execution of anoikis are mediated by different pathways, all of which ultimately converge into the activation of caspases and downstream molecular pathways 37. Although anoikis is mediated by multiple signaling pathways such as the Integrin-FAK, PI3K/Akt and MAPK pathways 23, 37, 38, studies have highlighted the crucial role of the EGFR pathway in conferring anoikis resistance to tumor cells in peripheral blood and facilitating metastasis 39-43. Our findings demonstrate that polyphyllin VII effectively inhibits the EGFR-Ras/Raf-MEK/ERK signaling pathway in CTC-TJH-01 and H1975 cells. Furthermore, our data reveal that upregulation of EGFR protein effectively counteracts the inhibitory effect of polyphyllin VII on CTC-TJH-01 cell migration. Moreover, the induction of anoikis by polyphyllin VII in CTC-TJH-01 cells was significantly reversed upon upregulation of the EGFR protein. These results highlight the important role of the EGFR pathway in mediating polyphyllin VII-induced anoikis and the inhibition of CTC migration. Other studies have reported that polyphyllin VII exhibits inhibitory effects on metastasis in different cancer cell types, such as HepG2 and MCF-7 cells, through the downregulation of the NF-κB/MMP-9/VEGF pathway or the inhibition of the EMT pathway 13, 44. Interestingly, our findings further support the notion that the EGFR pathway is instrumental in inducing anoikis of CTCs by polyphyllin VII, extending its relevance to lung cancer metastasis. In line with our* in vitro* observations, the administration of polyphyllin VII significantly impedes the lung metastasis of CTC-TJH-01 cells in NOD-SCID mice. Notably, the tumor cells within the lung metastatic nodules of polyphyllin VII-treated mice exhibited a more dispersed and less compact arrangement compared to those in the control group. Furthermore, the expression of EGFR protein was significantly downregulated in the lungs of mice treated with polyphyllin VII. Additionally, we observed a significant reduction in the number of CTC in the peripheral blood and the CTC number disseminated into the lungs of polyphyllin VII-treated mice. These *in vivo* results provide further evidence supporting the inhibitory effects of polyphyllin VII on CTC metastasis.

## Conclusion

In conclusion, our study provides evidence that polyphyllin VII exerts potent inhibitory effects on the migration and invasion of CTCs by inducing anoikis, both *in vitro* and *in vivo*. Notably, our findings highlight the crucial role of the EGFR-MEK/ERK pathway, rather than the BDNF/TrkB pathway, in mediating the induction of anoikis by polyphyllin VII in CTCs. These findings shed light on the therapeutic potential of polyphyllin VII as a promising agent for combating lung cancer metastasis. Further investigations are warranted to elucidate the precise underlying mechanisms and to assess the clinical efficacy of polyphyllin VII in the treatment of metastatic lung cancer.

## Abbreviations

CTCs: Circulating tumor cells
PFS: Progression-free survival
OS: Overall survival
TCM: Traditional Chinese medicine
EthD-1: Ethidium homodimer-1
GAPDH: Glyceraldehyde-3-phosphate dehydrogenase
FITC: Fluorescein isothiocyanate
PI: Propidium iodide
TrkB: Tyrosine kinase receptor B
BDNF: Brain-derived neurotrophic factor
EGFR: Epidermal growth factor receptor
MEK: Mitogen-activated protein kinase kinase
ERK1/2: Extracellular regulated protein kinases 1/2
